# Supplementary material for: Assessing Multi-Site rs-fMRI-Based Connectomic Harmonization Using Information Theory
Source: Brain Sci. 2022 Sep 9;12(9):1219. doi: 10.3390/brainsci12091219 (PMC9496818; doi:10.3390/brainsci12091219)
Supplement: Supplementary file 1 [file brainsci-12-01219-s001.zip › brainsci-1841637-supplementary.pdf]

Supplementary Material for Assessing Multi-Site rs-fMRI-Based Connectomic Harmonization Using Information Theory

Code

The code used in the elaboration of this work is available in the form of Jupiter Notebook at https://github.com/FacuRoffet99/HarmonizationEvaluation.

Figures

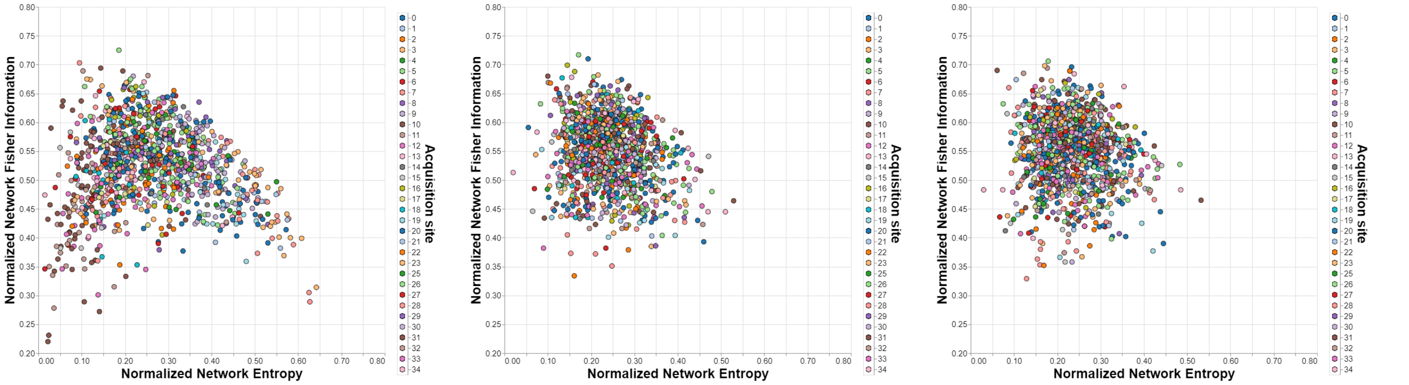

(a) Unharmonized (b) ComBat (c) CovBat

Figure S1: Shannon-Fisher planes for IMPAC/MSDL with different harmonization methods.

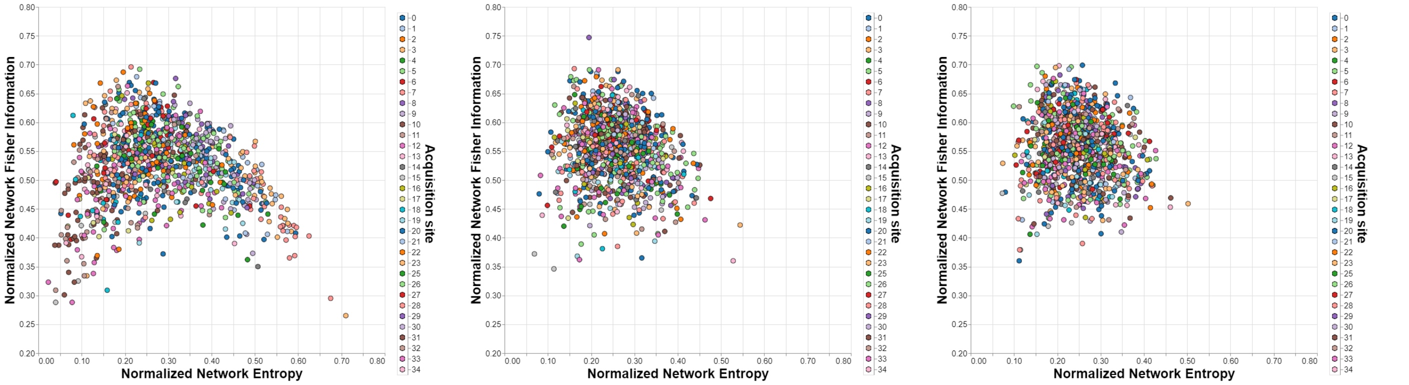

(a) Unharmonized (b) ComBat (c) CovBat

Figure S2: Shannon-Fisher planes for IMPAC/Harvard-Oxford with different harmonization methods.

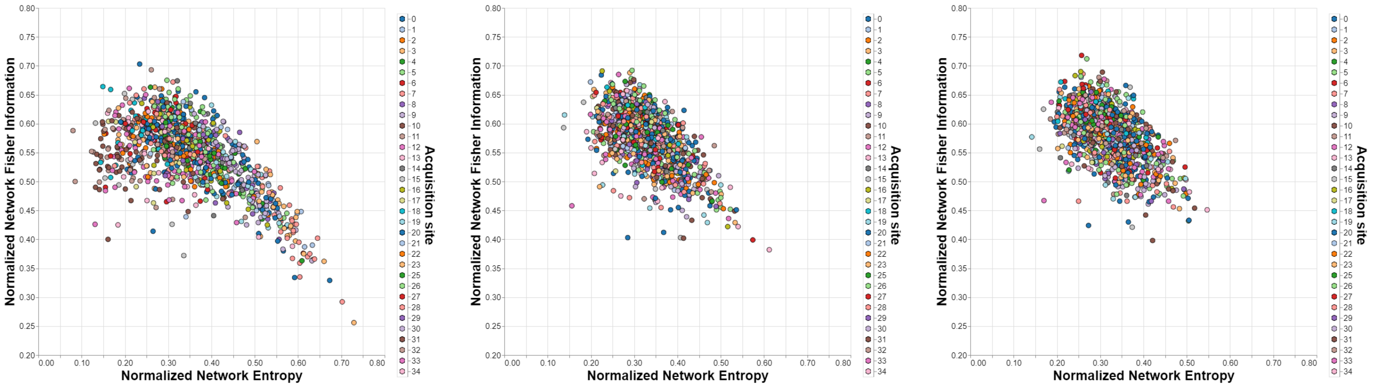

(a) Unharmonized (b) ComBat (c) CovBat

Figure S3: Shannon-Fisher planes for IMPAC/Basc064 with different harmonization methods.

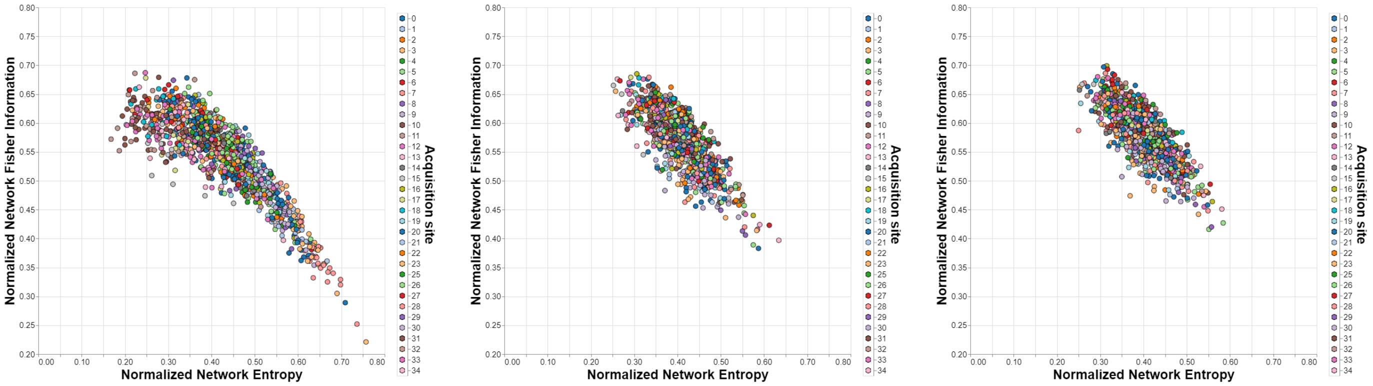

(a) Unharmonized (b) ComBat (c) CovBat

Figure S4: Shannon-Fisher planes for IMPAC/Basc122 with different harmonization methods.

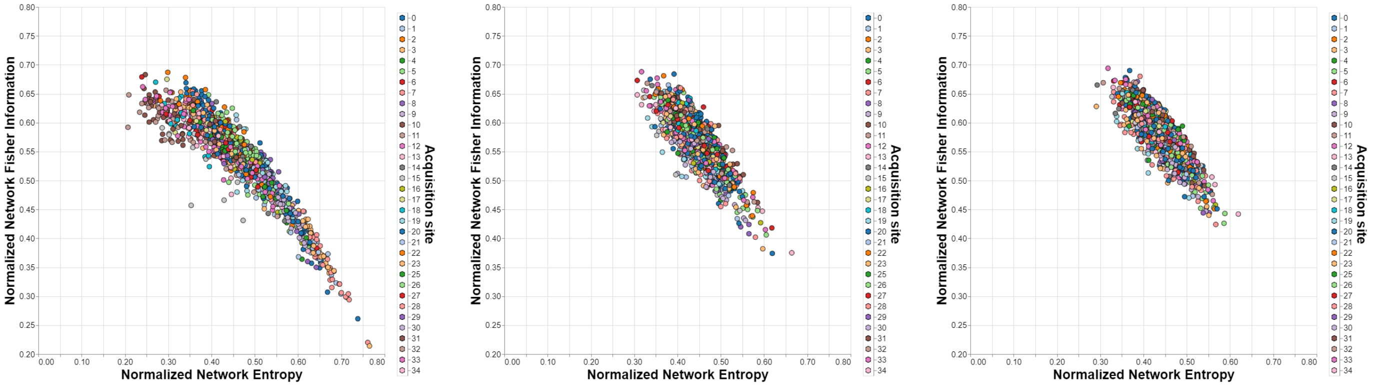

(a) Unharmonized (b) ComBat (c) CovBat

Figure S5: Shannon-Fisher planes for IMPAC/Basc197 with different harmonization methods.

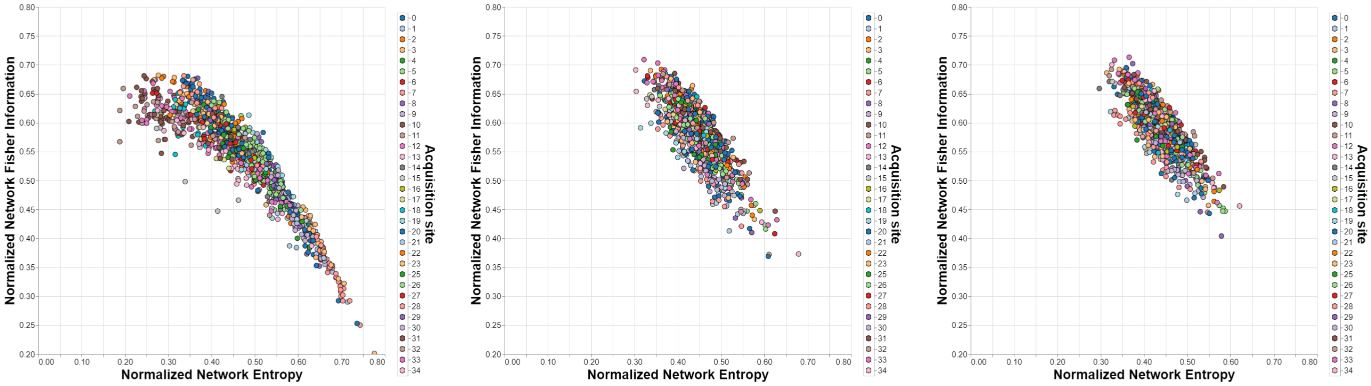

(a) Unharmonized (b) ComBat (c) CovBat

Figure S6: Shannon-Fisher planes for IMPAC/Craddock with different harmonization methods.

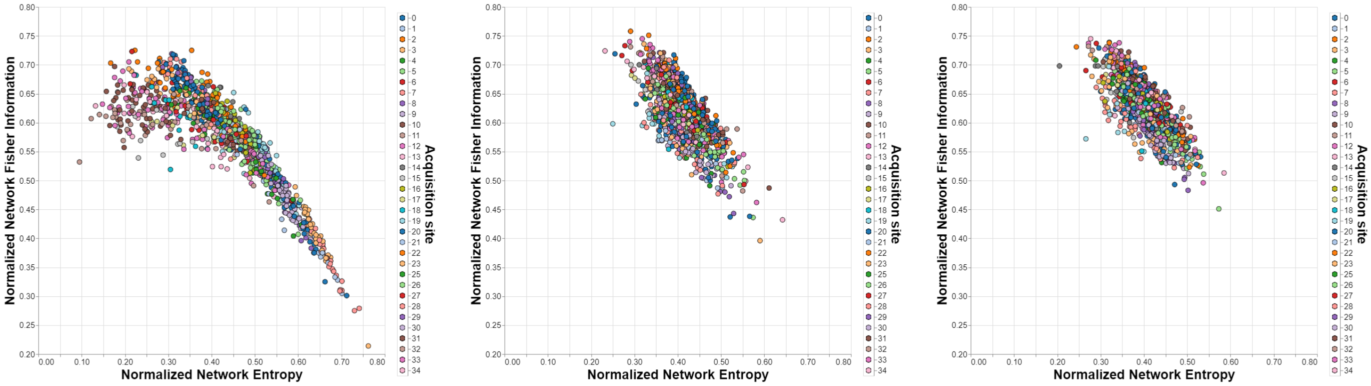

(a) Unharmonized (b) ComBat (c) CovBat

Figure S7: Shannon-Fisher planes for IMPAC/Power with different harmonization methods.

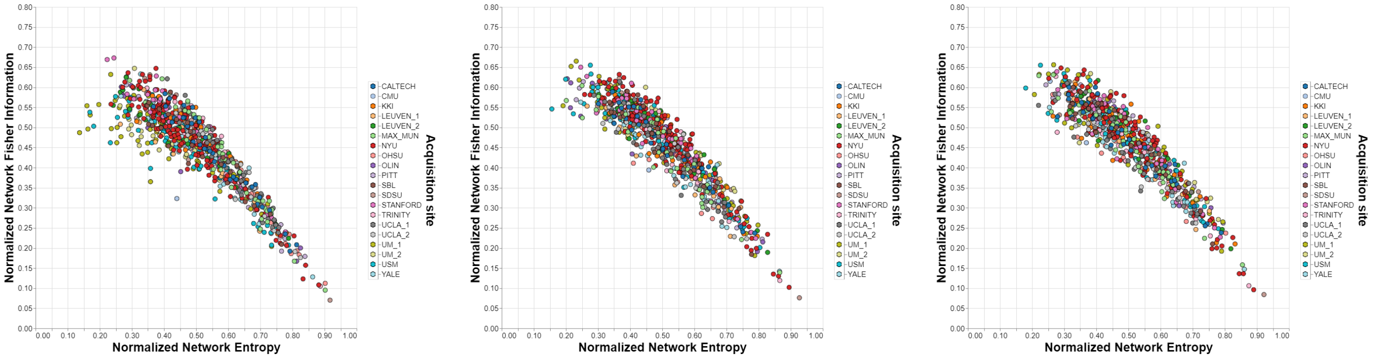

(a) Unharmonized (b) ComBat (c) CovBat

Figure S8: Shannon-Fisher planes for ABIDE/Talaraich-Tournoux with different harmonization methods.

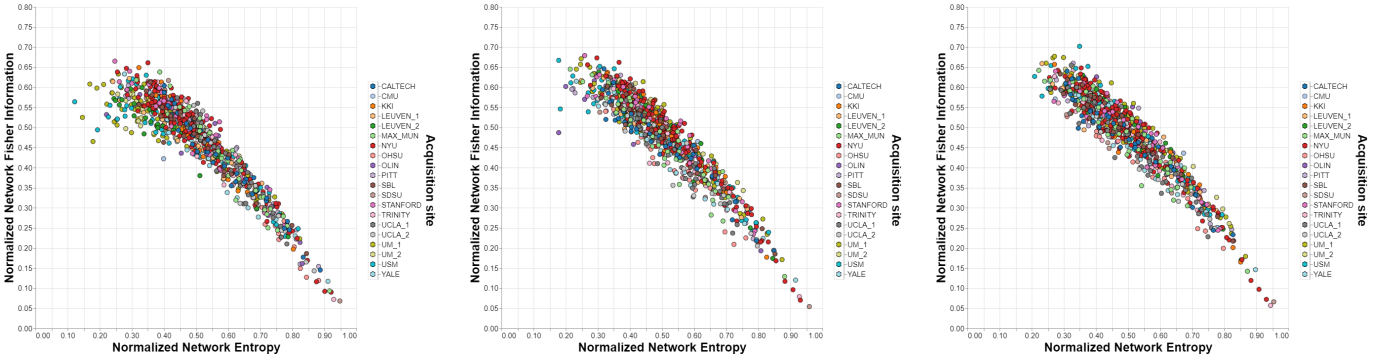

(a) Unharmonized (b) ComBat (c) CovBat

Figure S9: Shannon-Fisher planes for ABIDE/Harvard-Oxford with different harmonization methods.

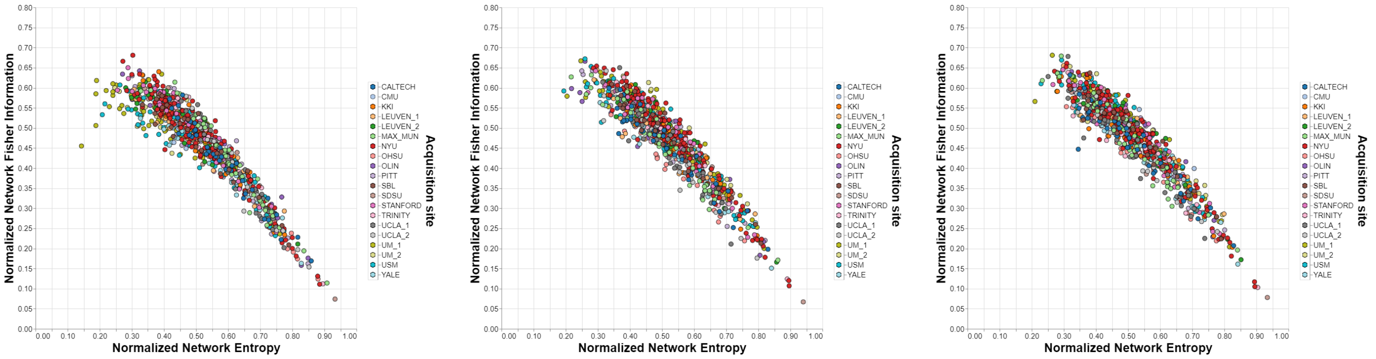

(a) Unharmonized (b) ComBat (c) CovBat

Figure S10: Shannon-Fisher planes for ABIDE/AAL with different harmonization methods.

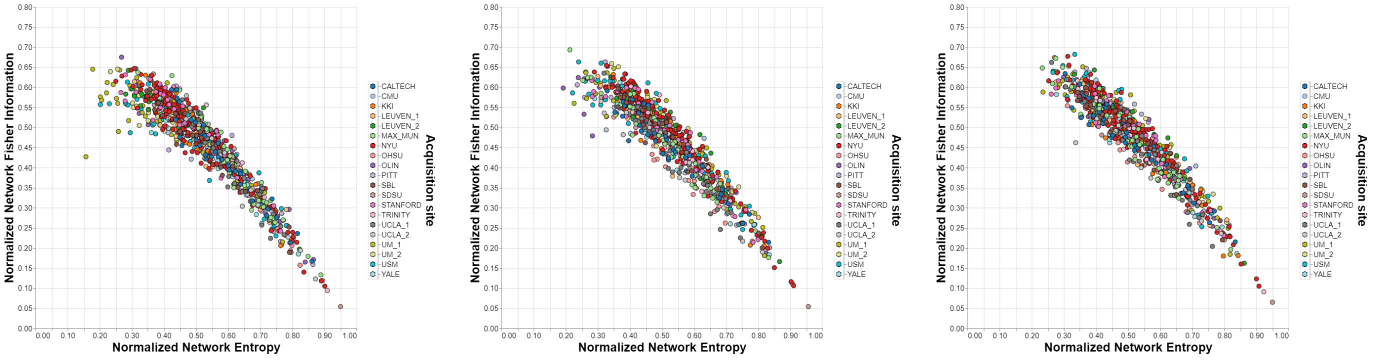

(a) Unharmonized (b) ComBat (c) CovBat

Figure S11: Shannon-Fisher planes for ABIDE/Eickhoff-Zilles with different harmonization methods.

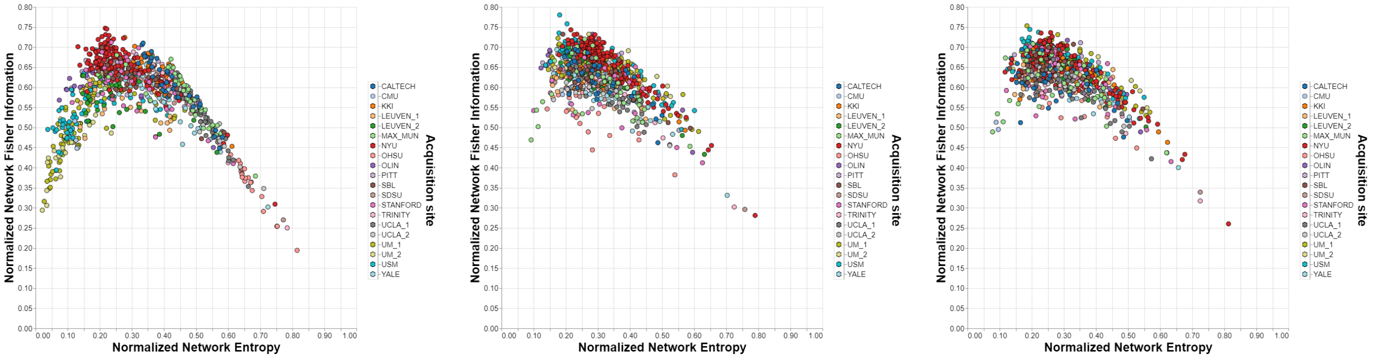

(a) Unharmonized (b) ComBat (c) CovBat

Figure S12: Shannon-Fisher planes for ABIDE/Dosenbach with different harmonization methods.

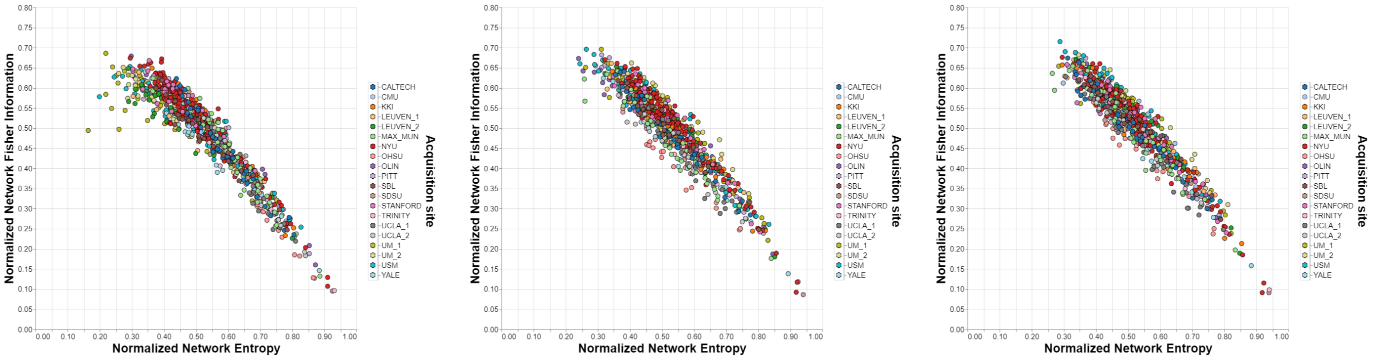

(a) Unharmonized (b) ComBat (c) CovBat

Figure S13: Shannon-Fisher planes for ABIDE/Craddock (200 ROIs) with different harmonization methods.

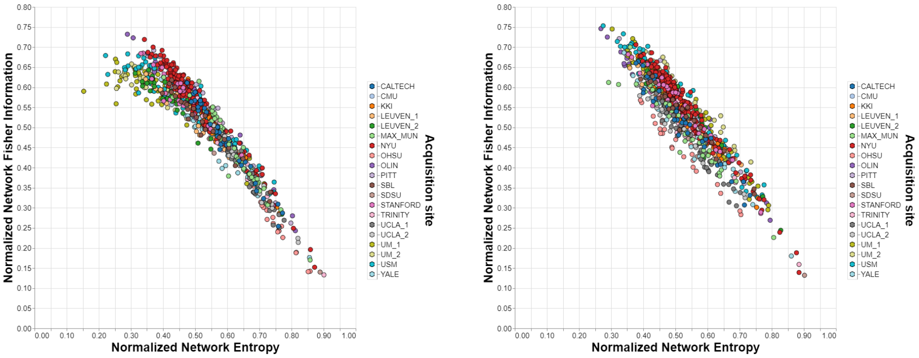

(a) Unharmonized (b) ComBat

Figure S14: Shannon-Fisher planes for ABIDE/Craddock (400 ROIs) with different harmonization methods.

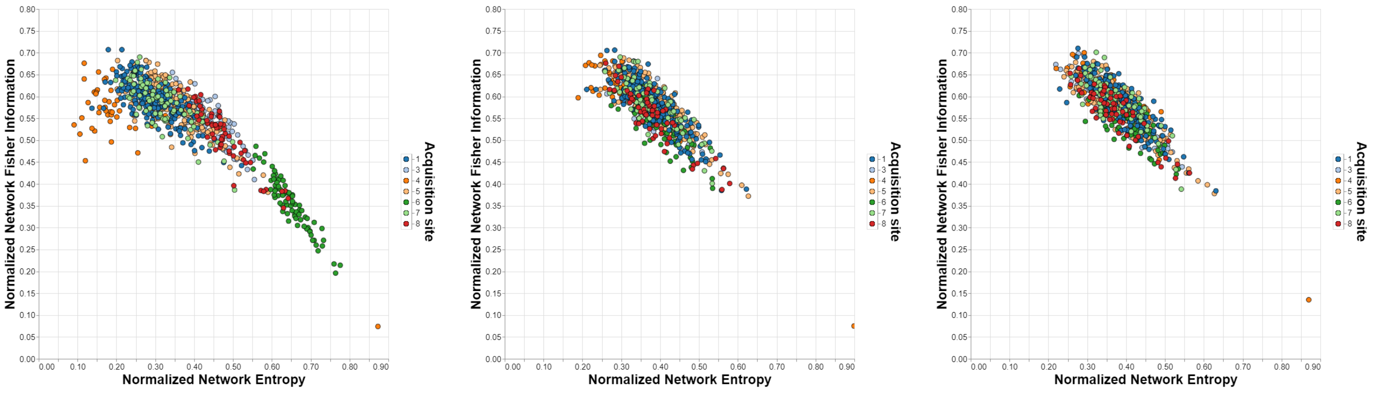

(a) Unharmonized (b) ComBat (c) CovBat

Figure S15: Shannon-Fisher planes for ADHD-200/Talaraich-Tournoux with different harmonization methods.

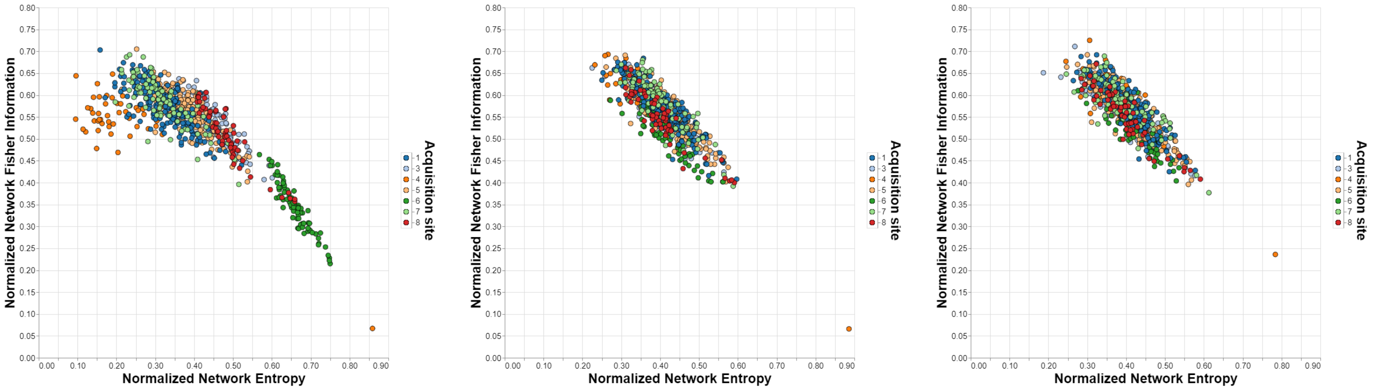

(a) Unharmonized (b) ComBat (c) CovBat

Figure S16: Shannon-Fisher planes for ADHD-200/Harvard-Oxford with different harmonization methods.

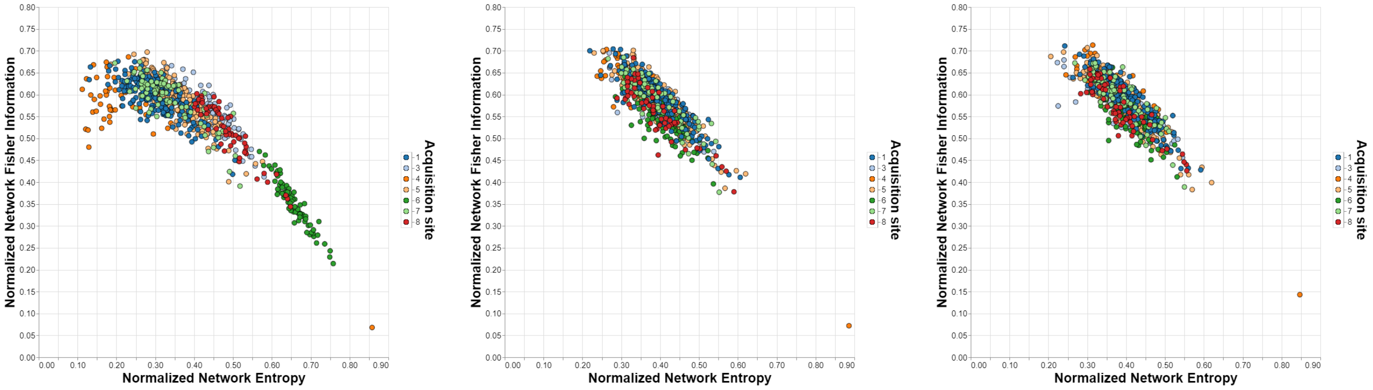

(a) Unharmonized (b) ComBat (c) CovBat

Figure S17: Shannon-Fisher planes for ADHD-200/AAL with different harmonization methods.

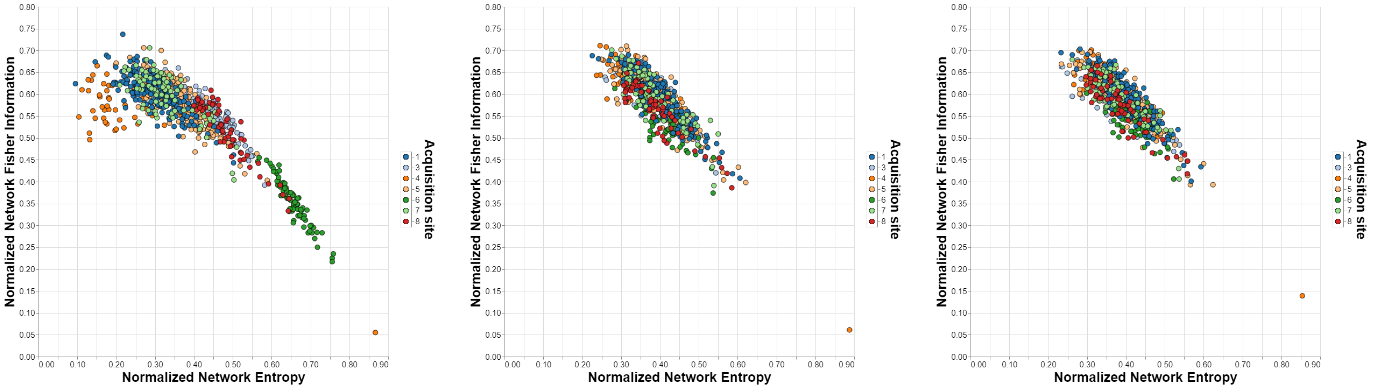

(a) Unharmonized (b) ComBat (c) CovBat

Figure S18: Shannon-Fisher planes for ADHD-200/Eickhoff-Zilles with different harmonization methods.

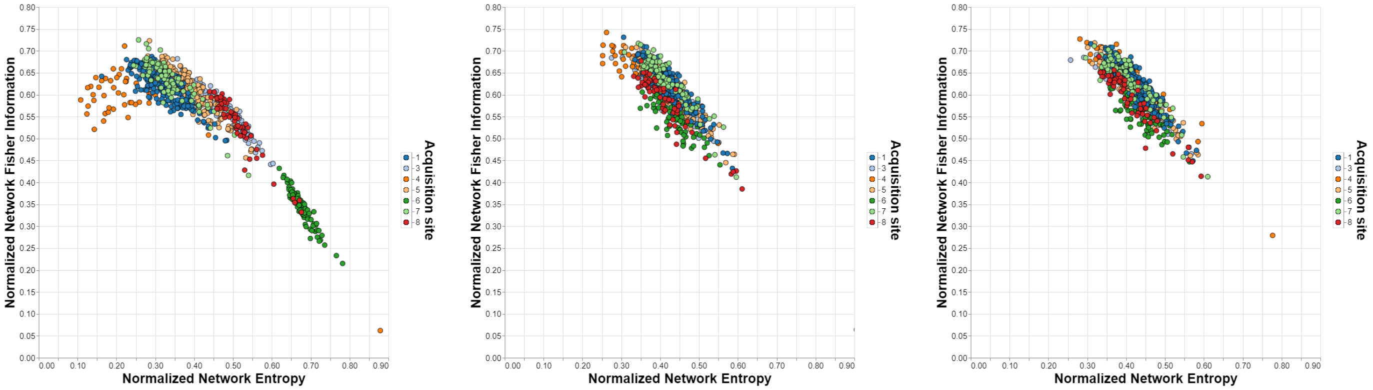

(a) Unharmonized (b) ComBat (c) CovBat

Figure S19: Shannon-Fisher planes for ADHD-200/Craddock (200 ROIs) with different harmonization methods.

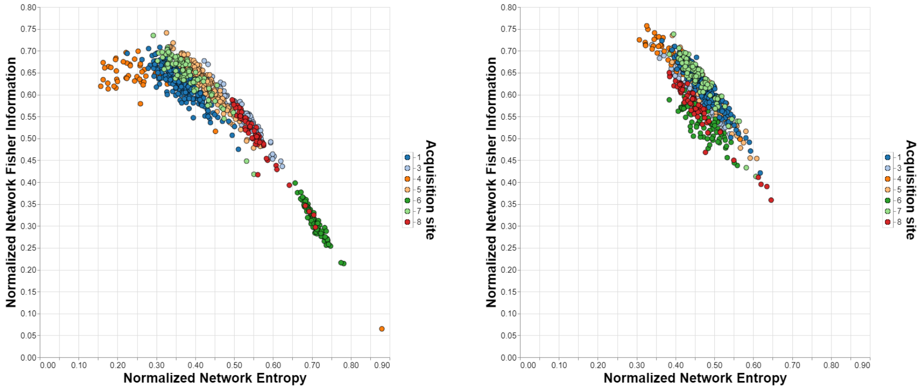

(a) Unharmonized (b) ComBat

Figure S20: Shannon-Fisher planes for ADHD-200/Craddock (400 ROIs) with different harmonization methods.

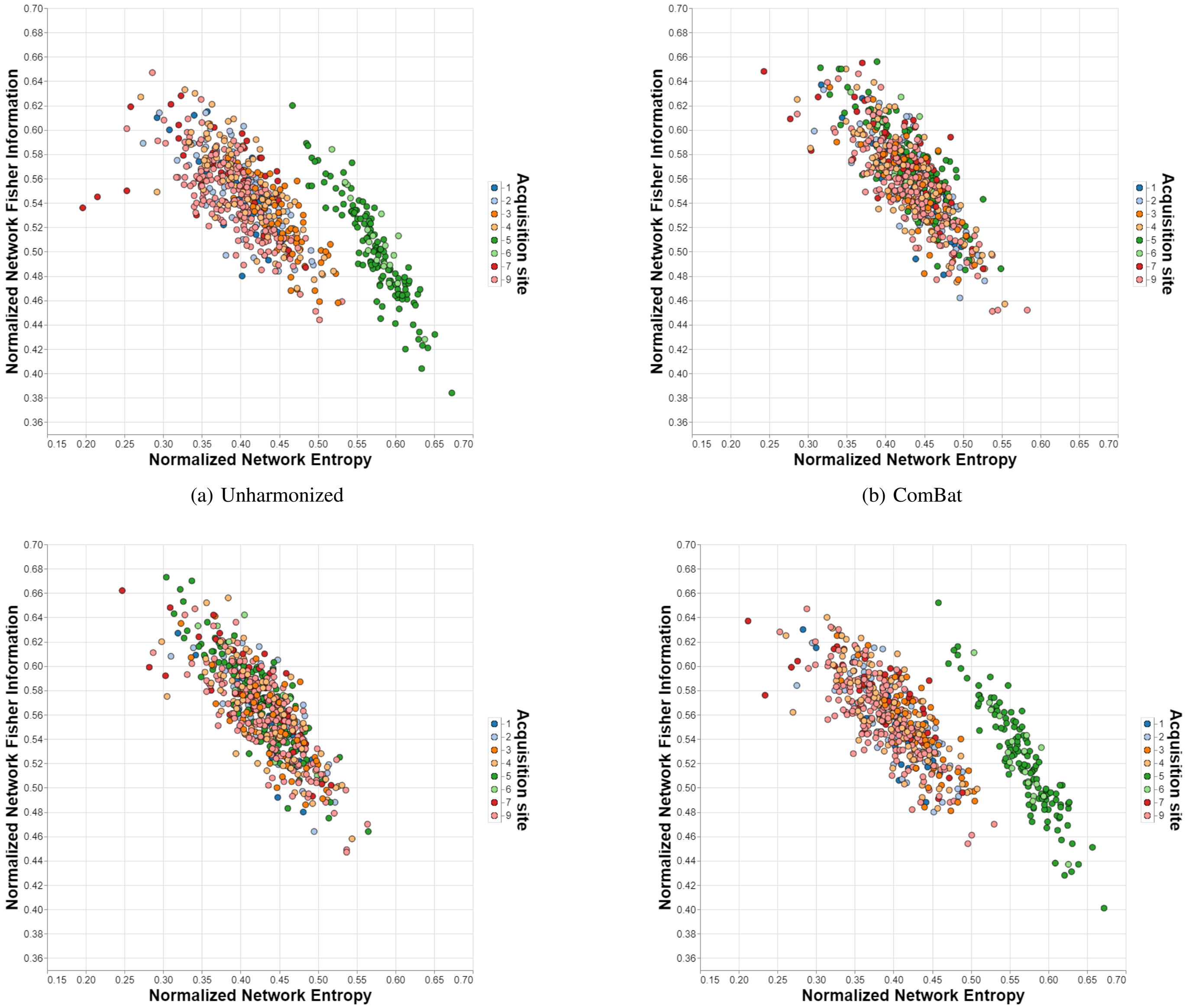

(a) Unharmonized

(b) ComBat

(c) CovBat

(d) Traveling-subject

Figure S21: Shannon-Fisher planes for SRPBS with different harmonization methods.
